# Mechanistic Insights into the Anti-Virulence Effects of *Viroelixir*, a Phenolic Blend from Green Tea and Pomegranate, on *Streptococcus mutans*

**DOI:** 10.3390/antibiotics15040406

**Published:** 2026-04-17

**Authors:** Manal Dahdah, Vijaykumar D. Nimbarte, Mahmoud Rouabhia, Yasmine Ettouil, Hawraa Issa, Latifa Koussih, Mikhlid H. Almutairi, Abdelhabib Semlali

**Affiliations:** 1 Groupe de Recherche en Écologie Buccale, Faculté de Médecine Dentaire, Université Laval, Québec, QC G1V 0A6, Canada; manal.dahdah.1@ulaval.ca (M.D.); mahmoud.rouabhia@fmd.ulaval.ca (M.R.); yasmine.et-touil.1@ulaval.ca (Y.E.); 2 Department of Pharmacy, Birla Institute of Technology and Science (BITS) Pilani, Hyderabad Campus, Dist. Medchal, Hyderabad 500078, India; 3 DREAM-CV Lab, Centre for Outcomes Research and Evaluation, Research Institute of the McGill University Health Centre, Montreal, QC H4A 3J1, Canada; 4 Département des Sciences Expérimentales, Université de Saint Boniface, Winnipeg, MB R2H 0H7, Canada; lkoussih@ustboniface.ca; 5 Department of Zoology, College of Science, King Saud University, P.O. Box 2455, Riyadh 11451, Saudi Arabia; malmutairi@ksu.edu.sa

**Keywords:** *Viroelixir*, phenolic blend from tea and pomegranate, *Streptococcus mutans*, antibacterial activity, virulence, liquid chromatography–mass spectrometry, biofilm, hemolytic activity, virulence gene expression, cytotoxicity

## Abstract

**Background**: Dental caries remains one of the most prevalent oral diseases worldwide, largely driven by the virulence of *Streptococcus mutans*. Although plant phenolics from green tea and pomegranate are known for their antimicrobial properties, their molecular mechanisms of action against key *S. mutans* virulence targets remain insufficiently characterized. **Aim**: This study investigated the antibacterial and anti-virulence properties of *Viroelixir*, a phenolic-rich formulation derived from green tea (*Camellia sinensis*) and pomegranate (*Punica granatum*), against *S. mutans*, with particular emphasis on predictive molecular docking interactions with critical virulence-associated proteins. **Methods**: *Viroelixir* phytochemical composition was characterized by LC–MS using a C18 reverse-phase column and negative electrospray ionization mode. Antibacterial activity was evaluated using growth kinetics, agar plating, and crystal violet assays. Acidogenicity, hemolytic activity, and biofilm formation were assessed using pH modulation, hemolysis assays, SEM, and biofilm biomass quantification. Virulence gene expression was analyzed by RT-qPCR. In silico molecular docking was performed to explore potential interactions between major LC–MS-supported phenolic constituents and *S. mutans* virulence proteins, including glucosyltransferase B (GtfB), LuxS, and SpaP. Biocompatibility was evaluated in human gingival epithelial cells. **Results**: The LC-MS analysis revealed a complex mixture of phenolic compounds consistent with catechins and ellagitannins. Compound identification was considered tentative and based on mass spectral range and chromatographic behavior. *Viroelixir* significantly inhibited *S. mutans* growth, acid production, hemolytic activity, and biofilm formation in a concentration-dependent manner. Key virulence genes were markedly downregulated. Docking analyses suggested stable binding of selected phenolics—particularly punicalagin, catechin, and epigallocatechin—within the active sites of *GtfB*, *LuxS*, and *SpaP*. Importantly, *Viroelixir* showed no cytotoxic effects on gingival epithelial cells. **Conclusions**: *Viroelixir* exerts potent antibacterial and anti-virulence effects against *S. mutans* through a multi-target mechanism combining transcriptional suppression and predictive molecular inhibition of virulence proteins, supporting its potential as a safe, natural therapeutic for caries prevention.

## 1. Introduction

Dental caries remains one of the most prevalent and persistent oral health problems worldwide, affecting both children and adults [1]. It is a multifactorial disease arising from the complex interplay among acidogenic bacteria, dietary carbohydrates, and host-related factors [2,3,4]. The microbial etiology of caries is now well established, with *Streptococcus mutans* (*S. mutans*) recognized as a principal causative agent in the initiation and progression of the disease [5,6]. According to the plaque hypothesis, *S. mutans* acts as a key driver of tooth decay through several virulence mechanisms, notably its ability to form biofilms, produce acids, and survive in low-pH environments [7,8,9].

The virulence of *S. mutans* is mediated mainly by glucosyltransferases (Gtfs) that synthesize extracellular polysaccharides (EPS), enhancing adhesion and promoting biofilm maturation, and retaining acids within the matrix, thereby accelerating enamel demineralization [10,11]. Glucan-binding proteins reinforce bacterial aggregation, while mutacins inhibit commensal competitors, facilitating ecological dominance [12,13,14]. These factors are regulated by the competence-stimulating peptide (CSP) via the ComDE two-component regulatory system [15], which also influences genetic competence through the ComRS–ComX pathway [16,17,18].

Adhesion is further mediated by the surface protein SpaP (P1), strongly associated with caries risk. Stress tolerance is supported by (p)ppGpp synthetases (*relA*, *relP*, *relQ*), which enable bacterial survival under nutrient limitation and acidity via the ComRS–ComX pathway [16,17,18]. Adhesion to the tooth surface also involves sucrose-independent mechanisms mediated by surface adhesins such as P1 (SpaP/AgI/II) [19,20,21,22,23]. The *spaP* gene has been strongly associated with caries risk, particularly in children [24]. Furthermore, *S. mutans* displays remarkable stress tolerance and survival ability under nutrient deprivation or acidic conditions, partly regulated by (p)ppGpp synthetases encoded by *relA*, *relP*, and *relQ* genes [25]. These enzymes mediate the stringent response, which enables bacterial persistence under unfavorable conditions.

Despite significant advances in understanding *S. mutans* virulence, current caries prevention strategies still rely heavily on conventional antimicrobials, including chlorhexidine and fluoride [26]. However, these approaches face several limitations: the rise in antimicrobial resistance, limited discovery of new antibiotic classes, and ecological disruption of the oral microbiota [27]. Chlorhexidine, although widely used, may induce resistance in *S. mutans* and alter microbial balance [28], while excessive fluoride exposure poses health risks [29,30]. Consequently, there is an urgent need for safer, more sustainable alternatives that effectively inhibit cariogenic bacteria without compromising oral ecology.

Natural products have gained growing attention as promising antimicrobial agents [31,32,33]. The World Health Organization has recognized the importance of phytotherapy in primary healthcare, advocating research into plant-derived compounds with therapeutic potential [34,35]. Among these, phenolics, a diverse group of plant secondary metabolites [34], exhibit potent antioxidant, anti-inflammatory, and antimicrobial properties [36,37,38,39]. Green tea (*Camellia sinensis*) and pomegranate (*Punica granatum*) are particularly rich in phenolic compounds, including catechins, tannins, ellagic acid, and anthocyanins, which have demonstrated antibacterial activity against oral pathogens [40]. Green tea catechins, especially epigallocatechin-3-gallate (EGCG) and epicatechin-3-gallate (ECG), are known to disrupt bacterial cell membranes [41] and inhibit glucosyltransferase activity, while pomegranate extracts reduce bacterial adhesion and modulate inflammatory responses in oral tissues [42,43].

Based on these complementary bioactivities, we developed *Viroelixir*, a novel formulation combining green tea and pomegranate extracts, designed to enhance antimicrobial efficacy while maintaining biocompatibility. The present study aimed to evaluate the antibacterial, anti-biofilm, and anti-virulence effects of *Viroelixir* against *S. mutans*, with a particular focus on its potential as a natural cavity disinfectant and preventive agent in restorative dentistry.

## 2. Results

### 2.1. Phytochemical Characterization of Viroelixir by LC–MS Analysis

**Figure 1:**
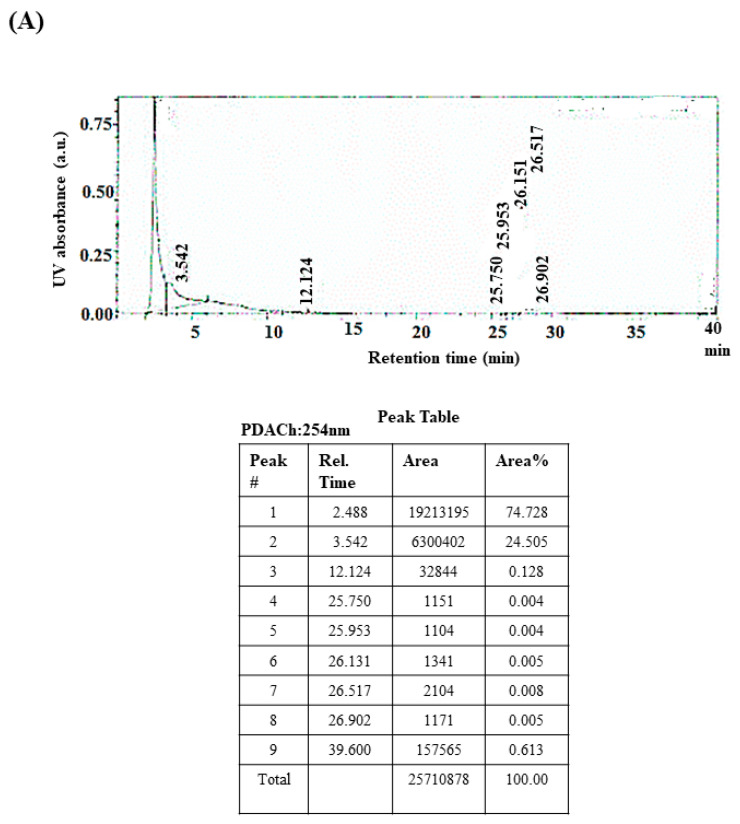

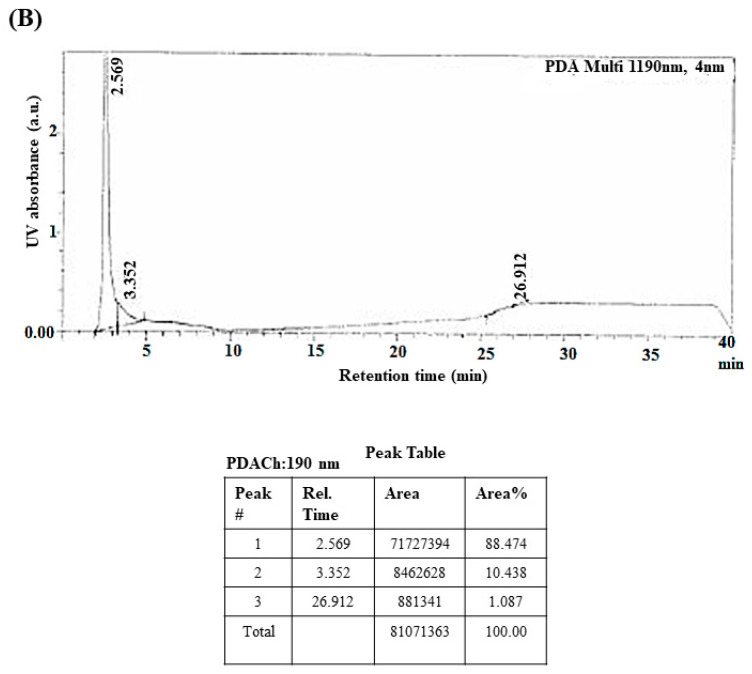

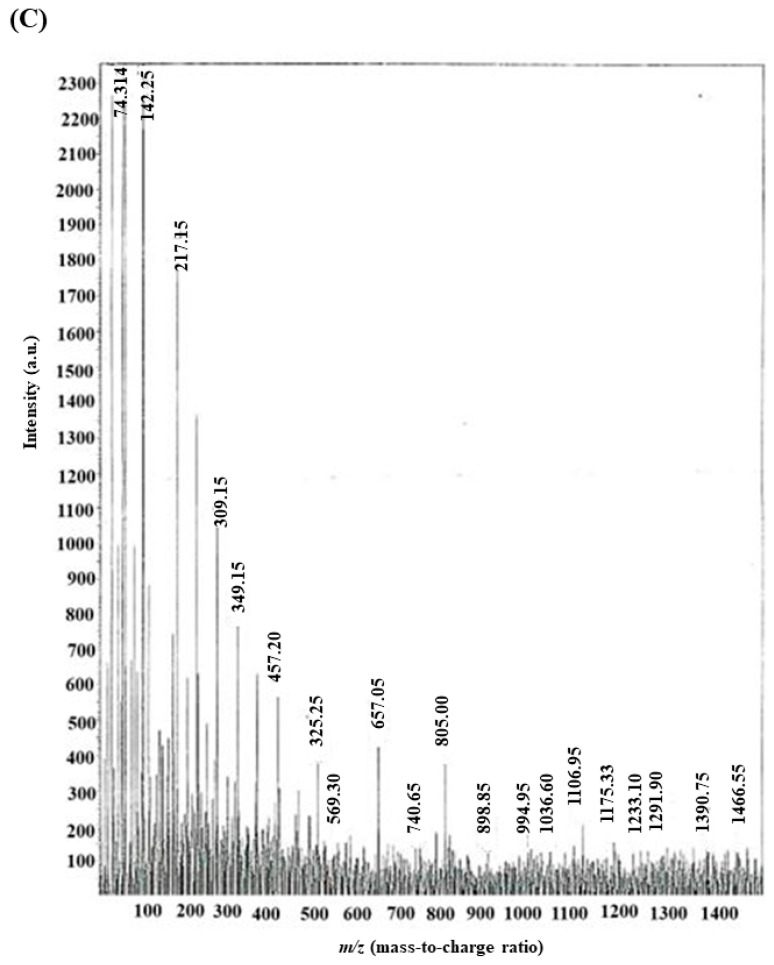
LC-MS analysis. (**A**) LC chromatogram recorded at 254 nm showing two major peaks at approximately 2.5 and 3.5 min. (**B**) LC chromatogram recorded at 190 nm, showing the same trend, and confirming the presence of two dominant compound families. (**C**) Total ion chromatogram (TIC) displaying a signal distribution between *m*/*z* 74 and 1400, consistent with the complex phenolic nature of the plant extract.

The phytochemical composition of *Viroelixir* was determined using liquid chromatography–mass spectrometry (LC–MS) to identify its major bioactive constituents and confirm its phenolic profile [44]. The UV chromatograms recorded at 254 nm and 190 nm revealed two dominant peaks eluting at approximately 2.5 min and 3.5 min, respectively (Figure 1A,B), indicating the predominance of two major phytochemical families. These peaks accounted for most of the chromatographic signal intensity, consistent with the presence of abundant phenolic compounds derived from green tea and pomegranate.

Mass spectrometry analysis revealed a broad molecular mass distribution ranging from *m*/*z* 74 to 1400 (Figure 1C), characteristic of structurally diverse phenolic compounds. Based on the observed mass range and the known phytochemical composition of green tea (*Camellia sinensis*) and pomegranate (*Punica granatum*), several major bioactive compounds were tentatively identified.

Compounds with molecular masses between *m*/*z* 290 and 460 are consistent with catechins originating from green tea, including catechin (*m*/*z* 290), epicatechin (*m*/*z* 290), epigallocatechin (*m*/*z* 306), epicatechin gallate (*m*/*z* 442), and epigallocatechin gallate (*m*/*z* 458), which represent the dominant antimicrobial constituents of green tea extracts. These compounds typically elute at early retention times due to their polar nature and likely correspond to the peak observed at approximately 2.5 min.

Higher molecular weight compounds detected between *m*/*z* ~600 and 1100 were consistent with hydrolysable ellagitannins such as punicalagin and punicalin. In contrast, lower molecular weight compounds, including gallic acid (*m*/*z* 170) and ellagic acid (*m*/*z* 302), were correctly classified as phenolic acids. Compound identification was considered tentative and based on mass spectral range, chromatographic behavior, and comparison with literature data. Definitive compound confirmation will require targeted MS/MS fragmentation and comparison with authentic standards. UV peak-area normalization provides qualitative and semi-quantitative information but does not reflect absolute compound abundance due to class-dependent molar absorptivity differences. Importantly, these compounds were further suggested through molecular docking analysis, where the major phenolics identified in *Viroelixir*—including punicalagin, punicalin, catechin, epigallocatechin, ellagic acid, and caffeic acid—were selected and structurally characterized. These identified compounds were subsequently used for in silico docking against key *Streptococcus mutans* virulence proteins, including glucosyltransferase B (GtfB), the quorum-sensing enzyme LuxS, and the adhesion protein SpaP.

Docking compounds were selected based on LC-MS profile consistency and literature-reported major polyphenols in green tea and pomegranate extracts. These analyses provide mechanistic hypotheses rather than definitive proof of direct molecular interaction. These docking results suggested potential interactions directly with virulence-associated proteins. This integrative approach combining LC–MS identification and molecular docking suggest a molecular basis that the phytochemical constituents of *Viroelixir* are responsible for its anti-virulence activity.

Collectively, the LC–MS analysis, supported by docking-based compound identification, confirms that *Viroelixir* contains a complex mixture of bioactive phenolics derived from green tea and pomegranate, predominantly including:

- Catechins (catechin, epicatechin, epigallocatechin, epigallocatechin gallate)
- Ellagitannins (punicalin)
- Phenolic acids (ellagic acid, gallic acid, caffeic acid)
- Flavonoid derivatives

These compounds are well known for their antibacterial, anti-biofilm, and anti-virulence activities and provide a molecular basis for the inhibitory effects of *Viroelixir* observed in subsequent biological and molecular experiments.

The association between chromatographic peaks and detected *m*/*z* signals was established by combining retention time behavior, accurate mass measurements, and comparison with previously reported phytochemical profiles of green tea and pomegranate extracts. Early-eluting peaks observed at approximately 2.5–3.5 min correspond to relatively polar phenolic compounds, particularly catechins and related flavonoids, which are known to elute rapidly under reversed-phase conditions. Lower *m*/*z* signals (≈290–460) are therefore consistent with catechin-type flavonoids, whereas higher *m*/*z* values detected in the spectra correspond to larger ellagitannin derivatives typically found in pomegranate extracts. Because MS/MS fragmentation and authentic standards were not used, these assignments should be considered tentative.

### 2.2. Antibacterial Activity of Viroelixir Against S. mutans

The antibacterial activity of *Viroelixir* against *S. mutans* was assessed using complementary assays evaluating growth kinetics, bacterial viability, and biomass formation.

**Figure 2:**
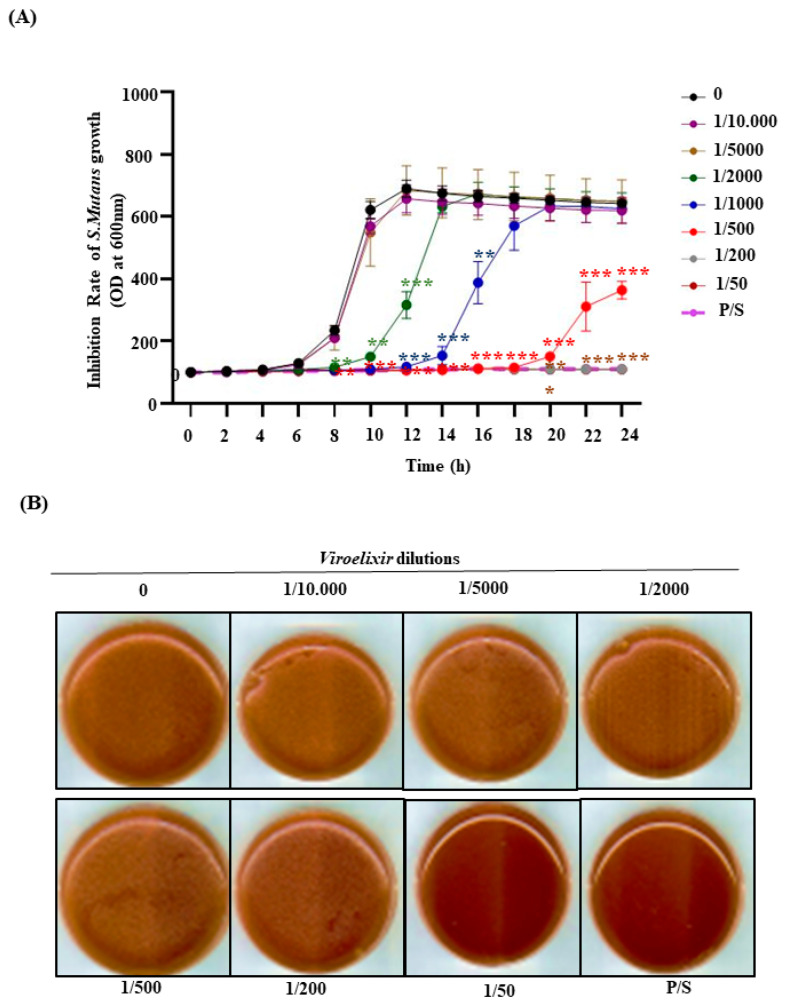

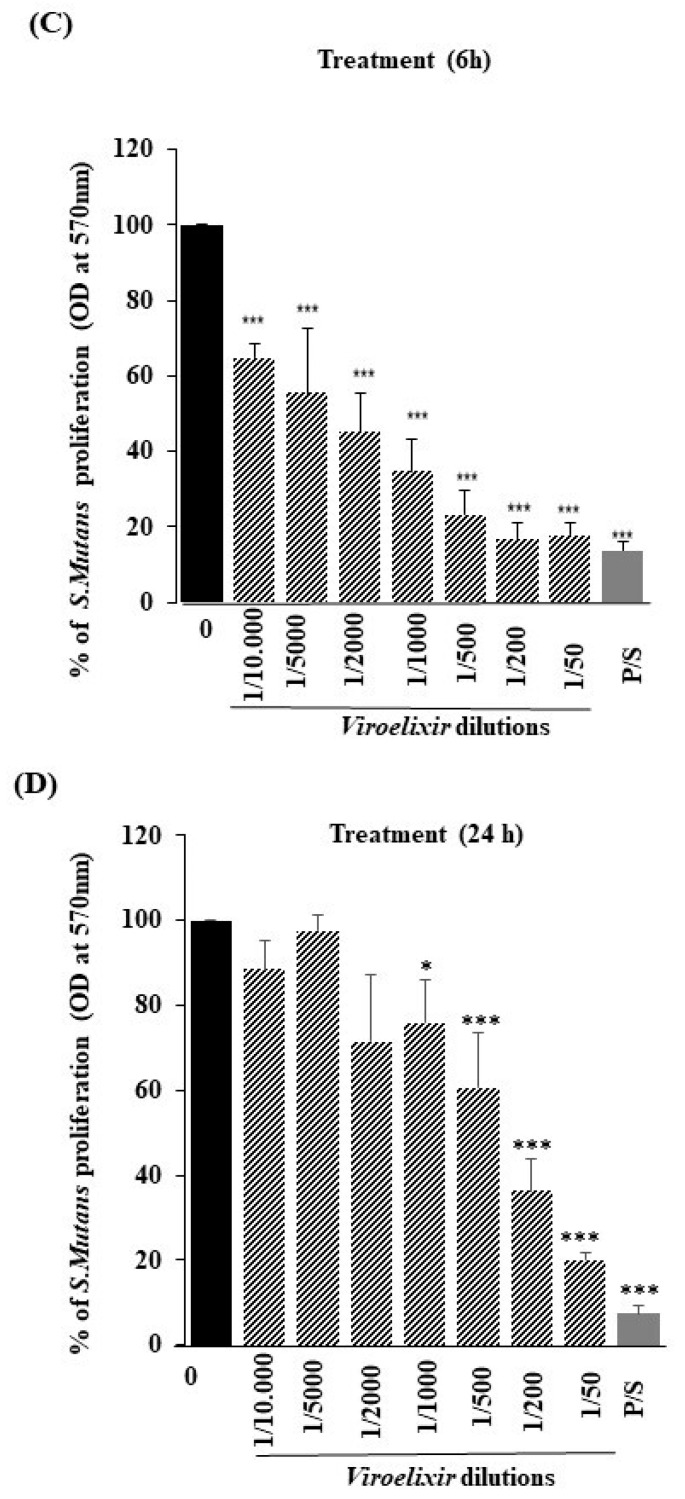
Inhibitory effect of *Viroelixir* on the growth of *S*. *mutans*. (**A**) Growth curve of *S. mutans*. Bacterial cells (10^5^ CFU/mL) were cultured in BHI broth and treated with *Viroelixir* at dilutions ranging from 1/10,000 to 1/50 at 37 °C for 24 h. The optical density at 600 nm (OD_600_) was recorded every 2 h. Dose-dependent growth inhibition was observed, with complete suppression at 1/50. The black, purple, brown, green, blue, red, gray, and dark red curves represent *Viroelixir* dilutions of 0, 1/10,000, 1/5000, 1/2000, 1/1000, 1/500, 1/200, and 1/50, respectively. The light purple curve represents S/P. (**B**) The BHI agar plate assay is used to determine the MBC. After 24 h incubation with *Viroelixir*, bacterial suspensions were plated on BHI agar. No colonies were detected at a 1/50 dilution, confirming the MBC, whereas higher dilutions showed partial or complete regrowth. (**C**,**D**) Crystal violet tests performed after 6 h (**C**) and 24 h (**D**) of treatment with *Viroelixir*. Biofilm biomass was quantified by measuring absorbance at 570 nm. Values are expressed relative to untreated *S. mutans* (set at 100%). The solid black bar represents the untreated control, the striped bars represent *Viroelixir*-treated groups at different dilutions, and the light gray bar represents the positive control (P/S). Data represent the mean ± SD of four independent experiments. (* *p* < 0.05, ** *p* < 0.005, *** *p* < 0.0005).

*Viroelixir* markedly inhibited *S. mutans* growth in a concentration- and time-dependent manner (Figure 2A). Complete growth suppression was observed at the 1/50 dilution, defining the minimum inhibitory concentration (MIC). At intermediate dilutions (1/200 and 1/500), *Viroelixir* induced a pronounced bacteriostatic effect during the early incubation phase, whereas sustained inhibition without regrowth was only achieved at the highest concentration.

To confirm whether growth inhibition reflected bactericidal activity, treated bacterial suspensions were plated on BHI agar. No colony formation was detected at the 1/50 dilution (Figure 2B), indicating that this concentration also corresponds to the minimum bactericidal concentration (MBC), while partial colony recovery was observed at higher dilutions.

Consistent with these findings, crystal violet staining revealed a significant reduction in bacterial biomass following *Viroelixir* treatment (Figure 2C,D). After 6 h, biomass was reduced by approximately 70% at 1/500 and by nearly 80% at 1/200 and 1/50. After 24 h, inhibition remained pronounced, exceeding 80% at the MIC/MBC concentration and remaining significant at intermediate dilutions.

Taken together, these results demonstrate that *Viroelixir* exerts potent antibacterial activity against *S. mutans*, with concordant inhibitory and bactericidal effects suggested across independent experimental approaches.

### 2.3. Inhibition of Acidogenicity in S. mutans

**Figure 3:**
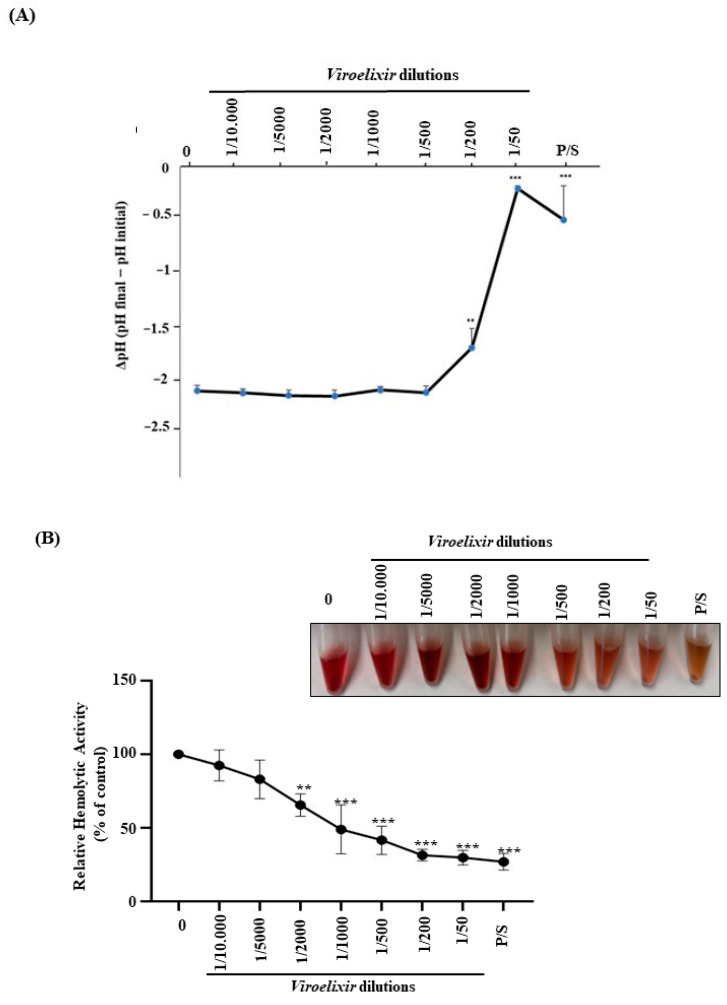
Effect of *Viroelixir* on hemolytic activity and pH modulation of *S. mutans*. (**A**) Measurement of ΔpH after 24 h of incubation. Untreated *S. mutans* showed strong acidification (ΔpH ≈ −2.09). *Viroelixir* reduced acid production in a dose-dependent manner, with partial inhibition at 1/200 (ΔpH = −1.69) and near-complete suppression at 1/50 (ΔpH = −0.21), comparable to the positive control. Statistical analysis showed significant differences relative to the untreated control (** *p* < 0.05, *** *p* < 0.005). (**B**) Relative hemolytic activity was measured after 24 h exposure to *Viroelixir* at 1/10,000, 1/5000, 1/2000, 1/1000, 1/500, 1/200, and 1/50, with P/S used as positive control. Untreated *S. mutans* displayed high hemolytic activity (100%). A significant dose-dependent reduction was observed starting at 1/2000 (*p* < 0.05), with further decreases at 1/1000 and 1/500 (*p* < 0.005 to *p* < 0.0005). At the highest concentrations (1/200 and 1/50), hemolytic activity dropped to approximately 20% of the control level, similar to the positive control. The intensity of the red coloration reflects the degree of hemolytic activity, with darker red indicating higher hemolysis.

The ability of *S. mutans* to produce acid is a key virulence trait contributing to enamel demineralization. As shown in Figure 3A, untreated *S. mutans* cultures exhibited marked acidification (ΔpH ≈ −2.09), indicative of active lactic acid production. *Viroelixir* treatment reduced acid production in a concentration-dependent manner. At high dilutions (1/10,000 to 1/500), ΔpH values remained close to those of the untreated control (≈−2.1), while a moderate inhibition was observed at 1/200 (ΔpH = −1.69). At the highest concentration (1/50), acid production was almost completely inhibited (ΔpH = −0.21), comparable to the positive control (ΔpH = −0.49). These findings indicate that *Viroelixir* effectively suppresses *S. mutans*’s acidogenic potential, thereby reducing its cariogenic capacity.

As shown in Figure 3B, the hemolytic activity of *S. mutans* was strongly affected by increased dilution of *Viroelixir*. Untreated *S. mutans* exhibited high hemolytic activity, set as 100% relative activity. Exposure to highly diluted *Viroelixir* (1/10,000 and 1/5000) produced only slight changes, indicating that low concentrations do not markedly alter hemolysin-related mechanisms. However, beginning at 1/2000, hemolytic activity significantly decreased and continued to drop with increasing *Viroelixir* concentration. At 1/1000 and 1/500, hemolytic activity was reduced by approximately 40–50%, with statistically significant differences compared with the untreated control. The strongest inhibition was observed at the highest concentrations (1/200 and 1/50), where hemolytic activity fell to around 20% of the control, reaching levels comparable to the positive control (P/S). These findings demonstrate that *Viroelixir* markedly suppresses the hemolytic potential of *S. mutans*, indicating an inhibitory effect on extracellular virulence factors involved in host cell damage and cytotoxicity.

### 2.4. Inhibition of Biofilm Formation

**Figure 4:**
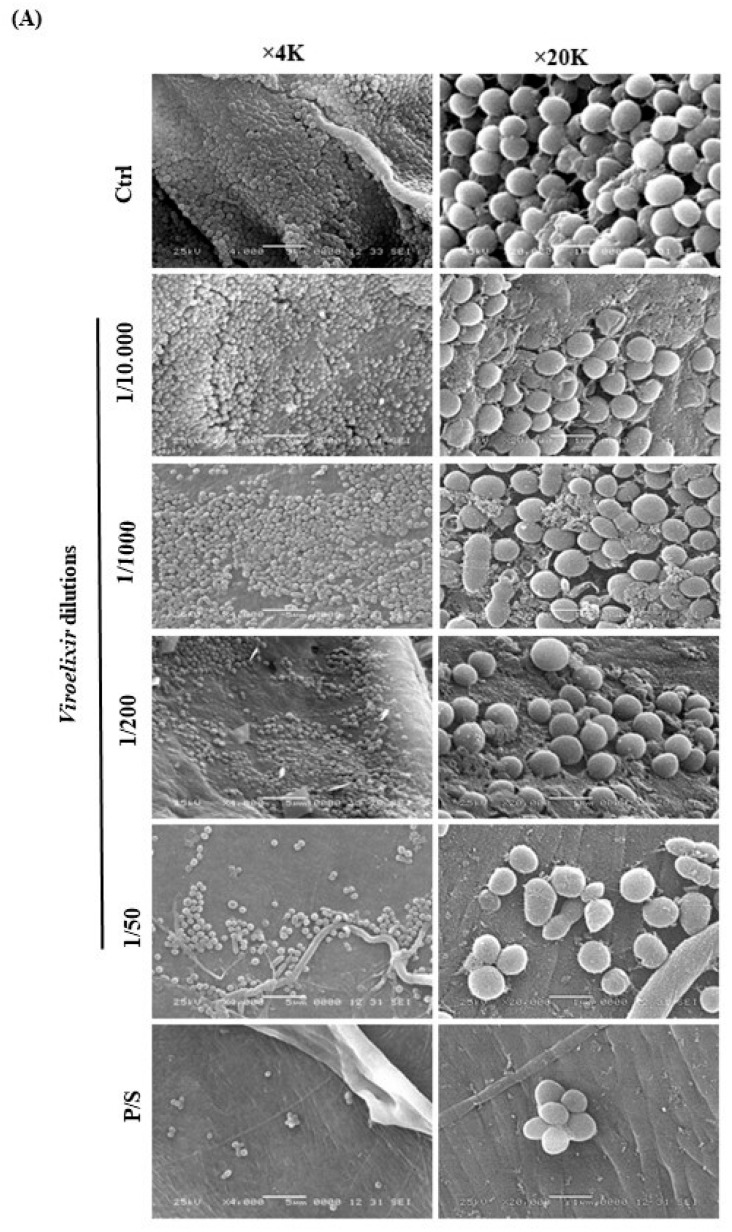

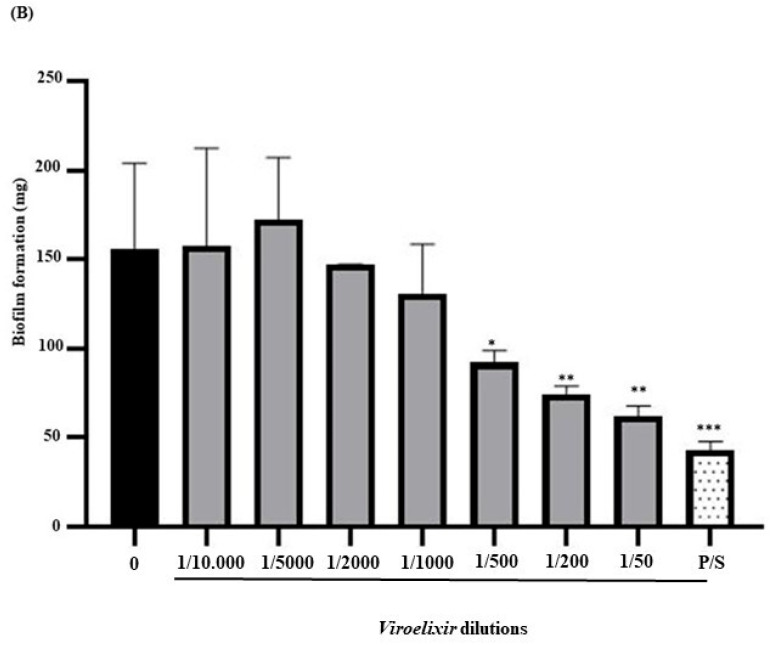
Effect of *Viroelixir* on biofilm formation of *S. mutans*. (**A**) Quantification of 6 day biofilm biomass by weight on porous membrane scaffolds following exposure to increasing dilutions of *Viroelixir*. P/S served as the positive control, and comparisons are presented relative to untreated controls. All data are expressed as mean ± SEM. * *p* < 0.05, ** *p* < 0.005, and *** *p* < 0.0005 were considered statistically significant. (**B**) Scanning electron microscopy images (*n* = *3*) showing 6 days *S. mutans* biofilm development on porous membrane scaffolds after exposure to *Viroelixir* or the positive control (P/S). Comparisons were made relative to untreated controls. The solid black bar represents the untreated control (0), the gray bars represent *Viroelixir*-treated groups at different dilutions, and the dotted bar represents the positive control (P/S).

Biofilm biomass quantification and morphological analyses were used to evaluate the anti-biofilm activity of *Viroelixir*. Quantitative measurements (Figure 4B) revealed a significant dose-dependent reduction in biofilm formation. Compared with untreated controls, biofilm mass decreased by approximately 50% at the 1/200 dilution (*p* < 0.05) and by ≈63% at the 1/50 dilution (*p* < 0.005), values comparable to those of the antibiotic control.

SEM observations (Figure 4A) suggested these findings. Untreated biofilms displayed dense, multilayered structures of coccoid cells embedded in a thick extracellular polymeric substance (EPS) matrix. *Viroelixir* treatment progressively disrupted this architecture: at 1/1000, clusters appeared less compact with reduced EPS; at 1/200, surface coverage decreased substantially; and at 1/50, only a few scattered or lysed cells remained, with minimal EPS. These data demonstrate that *Viroelixir* strongly interferes with bacterial adhesion and EPS synthesis, thereby preventing mature biofilm development.

### 2.5. Downregulation of S.mutans Virulence Gene Expression

**Figure 5:**
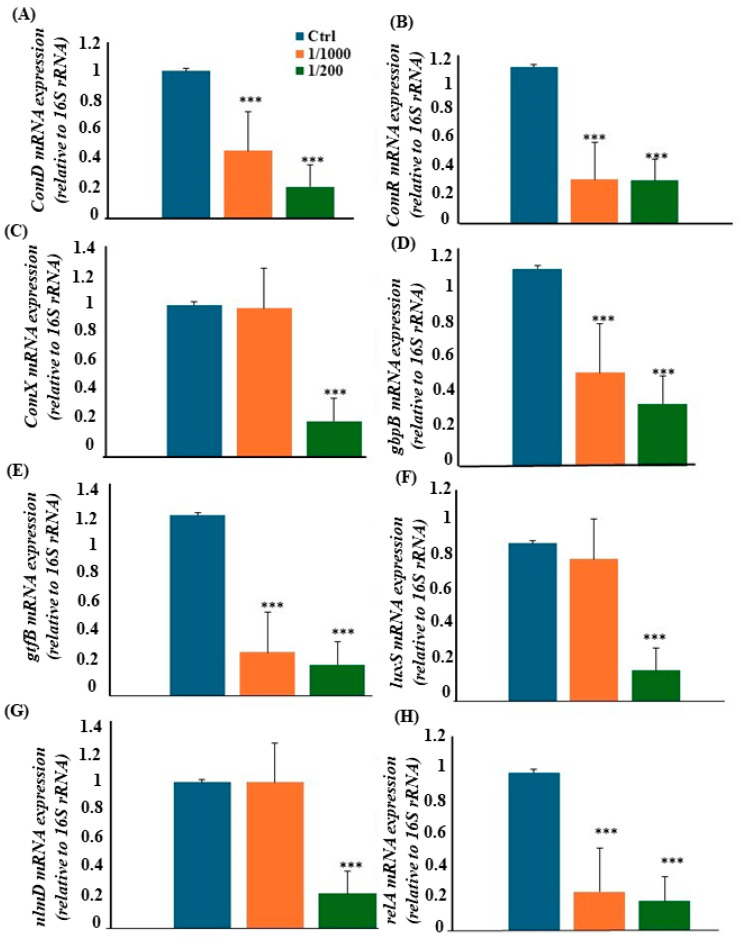

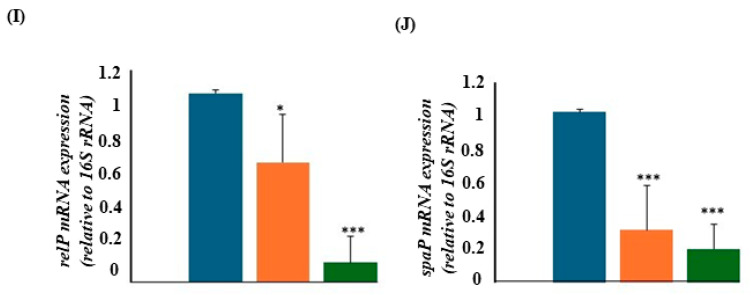
*Viroelixir* reduces virulence-related gene expression in *S. mutans*. *S. mutans* cells were treated with *Viroelixir* at 1/1000 and 1/200 for 24 h at 37 °C. Total RNA was extracted, reverse transcribed, and analyzed by qRT-PCR (*n* = 4). Expression levels were normalized to the 16S rRNA gene and expressed as fold changes relative to untreated cells. (**A**) comD expression; (**B**) comR expression; (**C**) comX expression; (**D**) gbpB expression; (**E**) gtfB expression; (**F**) luxS expression; (**G**) nlmD expression; (**H**) relA expression; (**I**) relP expression; (**J**) spaP expression. Expression was normalized to the 16S rRNA gene, which served as housekeeping control. * *p* < 0.05, *** *p* < 0.005.

To investigate the molecular basis of *Viroelixir*’s anti-virulence effects, the expression of ten major *S. mutans* genes associated with quorum sensing, adhesion, biofilm formation, and stress tolerance was analysed by RT-qPCR. As shown in Figure 5, all tested genes were significantly downregulated in *S. mutans* treated with *Viroelixir* (1/200 dilution) compared with untreated controls (*p* < 0.001).

Genes encoding quorum-sensing regulators (*comR*, *comD*, *comX*) were suppressed by approximately fivefold, indicating inhibition of the *ComRS–ComDE* signalling cascades. Similarly, *gbpB* (glucan-binding protein) and gtfB (*glucosyltransferase*) showed 2–5-fold reductions, consistent with impaired EPS synthesis and biofilm formation. The quorum-sensing genes *luxS* and *nlmD* were also significantly downregulated at 1/200, while stress-response genes (*relA*, *relP*) and the adhesin gene *spaP* showed ≈fivefold decreases. Collectively, these results demonstrate that *Viroelixir* markedly suppresses multiple virulence pathways, thereby weakening *S. mutans*’s overall pathogenic potential.

### 2.6. Molecular Docking Reveals Direct Targeting of Key Virulence Proteins

**Figure 6:**
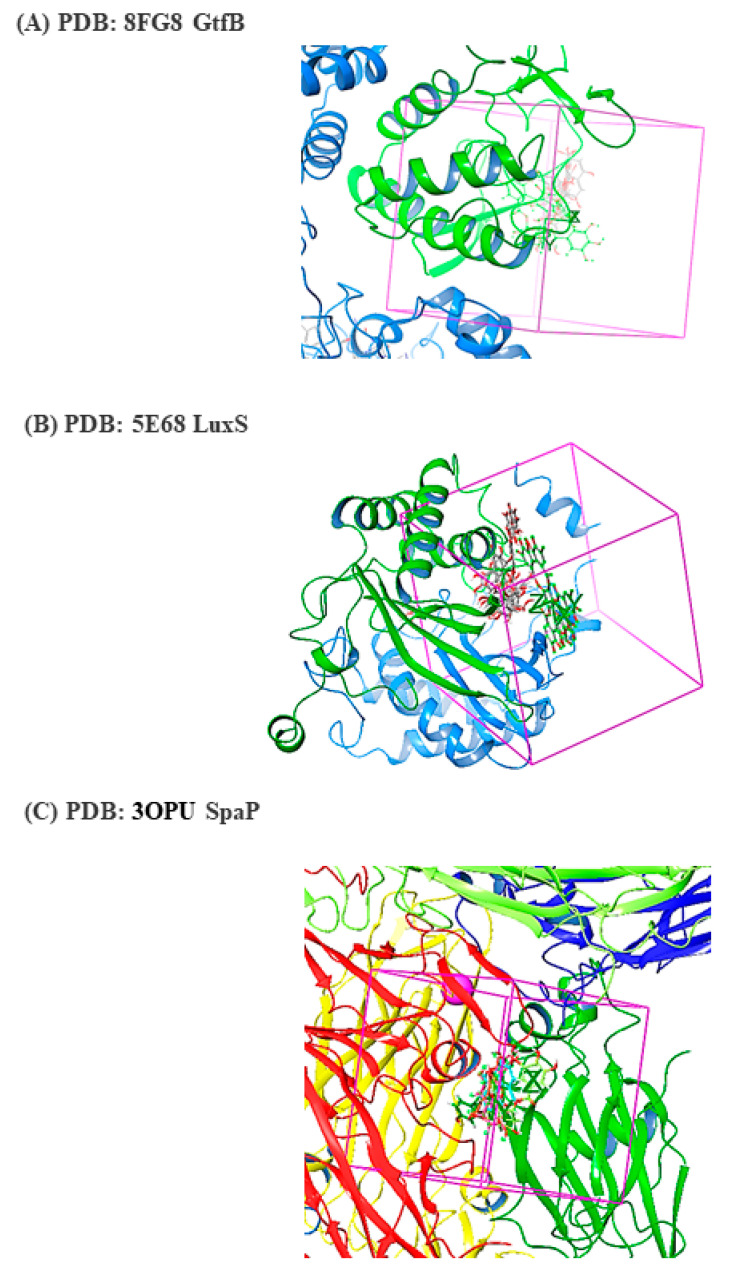
Docking simulations within the magenta grid box illustrate optimal ligand orientations at the predicted active sites. The protein structure is depicted in green (cartoon representation), while electrostatic surface mapping is displayed, with red indicating negative potential and blue indicating positive potential, to highlight key protein–ligand interactions. (**A**) Ligand docking within *S. mutans* glucosyltransferase B (GtfB). (**B**) Ligand binding to the LuxS quorum-sensing enzyme (PDB ID: 5E68). (**C**) Ligand interactions with the surface adhesin SpaP (PDB ID: 3OPU).

**Figure 7:**
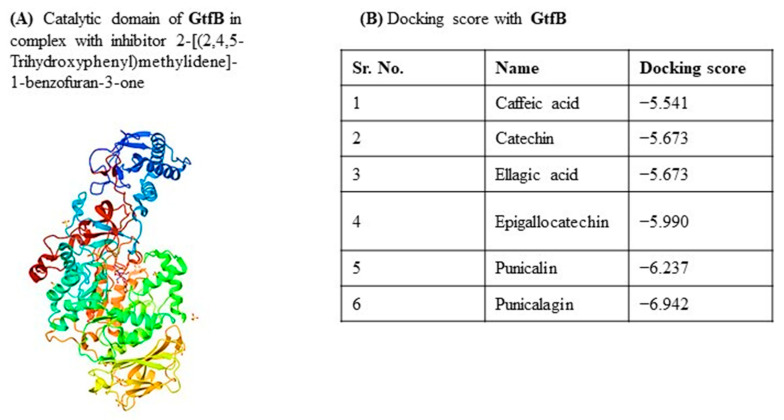

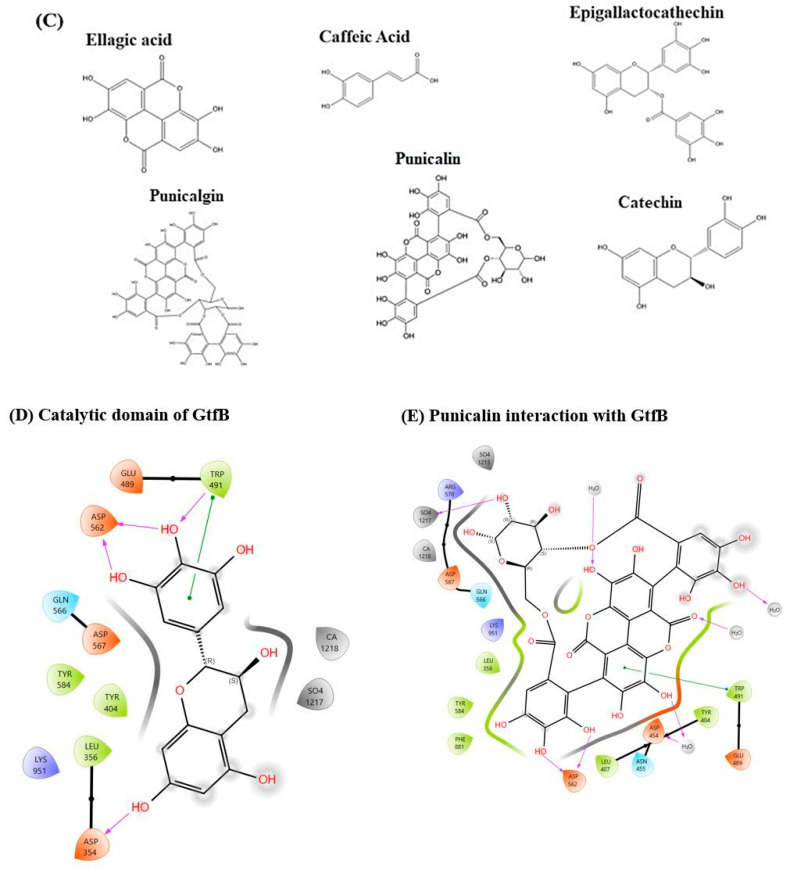

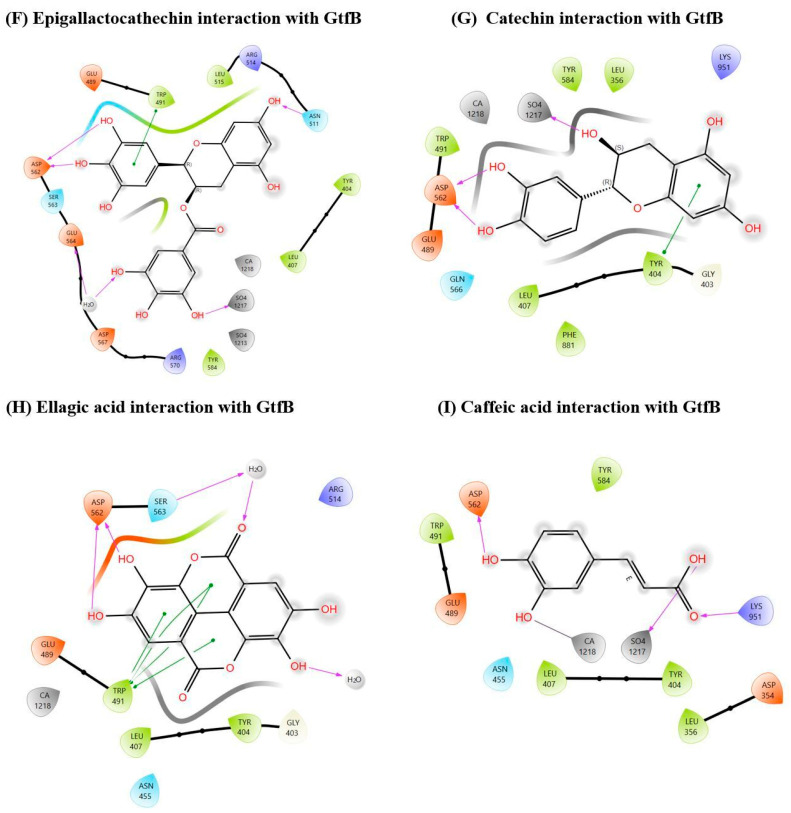
*Viroelixir* compounds docking with *Streptococcus mutans* Glucosyltransferase B (*Gtfb*). (**A**) Crystal structure of GtfB. The protein is shown in ribbon (cartoon) representation with a color gradient (blue to red) indicating the N- to C-terminal organization of the structure. (**B**) Docking scores of *Viroelixir* compounds with GtfB. (**C**) Chemical structures of *Viroelixir* compounds. (**D**) Catalytic domain of GtfB. The protein–ligand interaction is shown, with red labels indicating oxygen atoms and hydroxyl (–OH) groups involved in hydrogen bonding interactions. Arrows indicate the interactions between the ligand and key amino acid residues within the catalytic site. (**E**) Punicalin interaction with GtfB. The ligand–protein interaction map highlights key binding residues. Red labels indicate oxygen atoms and hydroxyl (–OH) groups involved in hydrogen bonding interactions. (**F**) Epigallocatechin interaction with GtfB. Red labels indicate oxygen atoms and hydroxyl (–OH) groups involved in hydrogen bonding interactions. Arrows represent hydrogen bonding and other non-covalent interactions between the ligand and amino acid residues. (**G**) Catechin interaction with GtfB. Red labels indicate oxygen atoms and hydroxyl (–OH) groups involved in hydrogen bonding interactions. Arrows represent hydrogen bonding and other non-covalent interactions between the ligand and amino acid residues. (**H**) Ellagic acid interaction with GtfB. Red labels indicate oxygen atoms and hydroxyl (–OH) groups involved in hydrogen bonding interactions. Arrows represent hydrogen bonding and other non-covalent interactions between the ligand and amino acid residues. (**I**) Caffeic acid interaction with GtfB. Red labels indicate oxygen atoms and hydroxyl (–OH) groups involved in hydrogen bonding interactions. Arrows represent hydrogen bonding and other non-covalent interactions between the ligand and amino acid residues.

**Figure 8:**
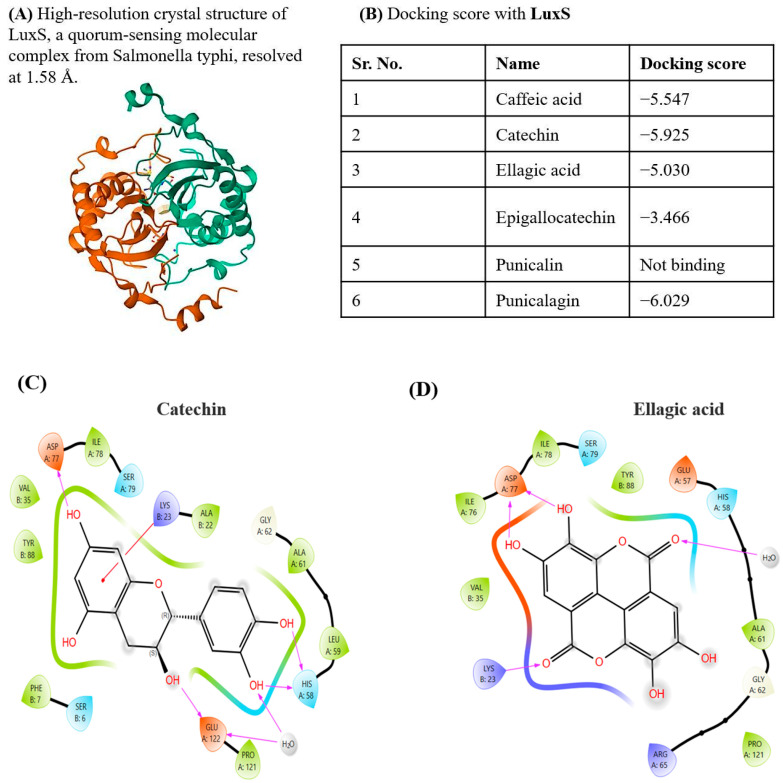

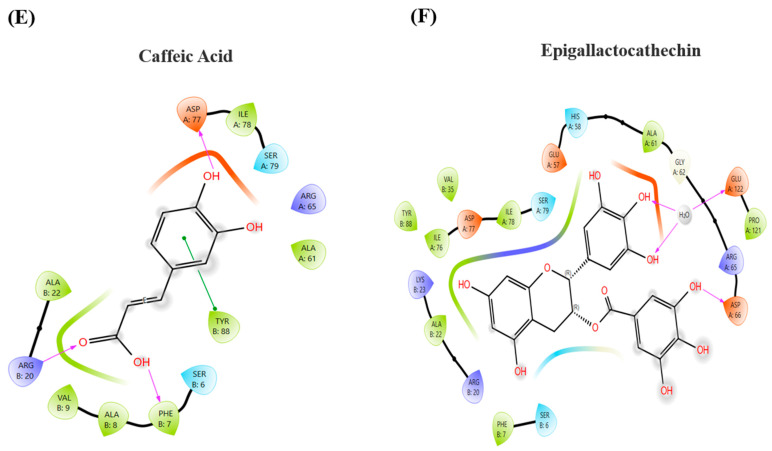
*Viroelixir* compounds docking with *Streptococcus mutans LuxS*. (**A**) High-resolution crystal structure of LuxS. The protein is shown in ribbon (cartoon) representation with a color gradient indicating its structural organization. (**B**) Docking scores of *Viroelixir* compounds with LuxS. (**C**) Interaction of catechin with LuxS. (**D**) Interaction of ellagic acid with LuxS. (**E**) Interaction of caffeic acid with LuxS. (**F**) Interaction of epigallocatechin with LuxS. Red labels indicate oxygen atoms and hydroxyl (–OH) groups involved in hydrogen bonding interactions. Arrows represent hydrogen bonding and other non-covalent interactions between ligands and amino acid residues. Colored contours and lines illustrate different interaction environments within the binding pocket.

**Figure 9:**
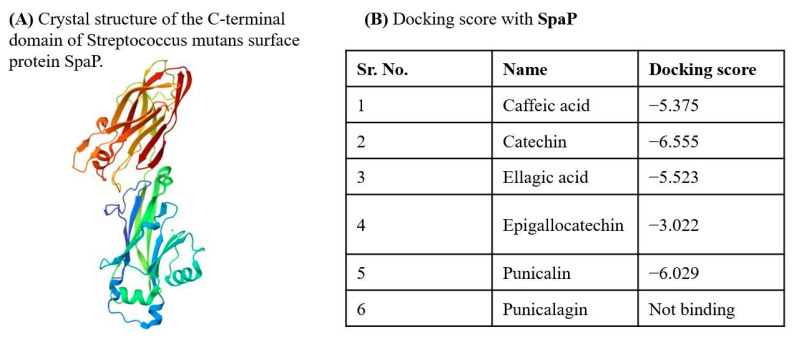

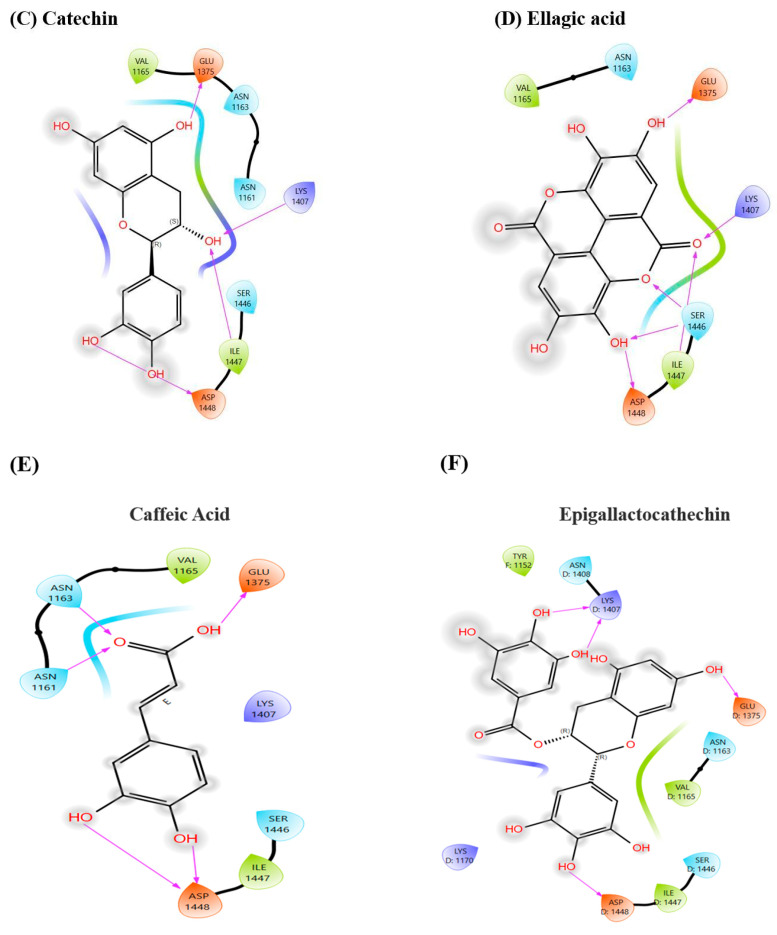

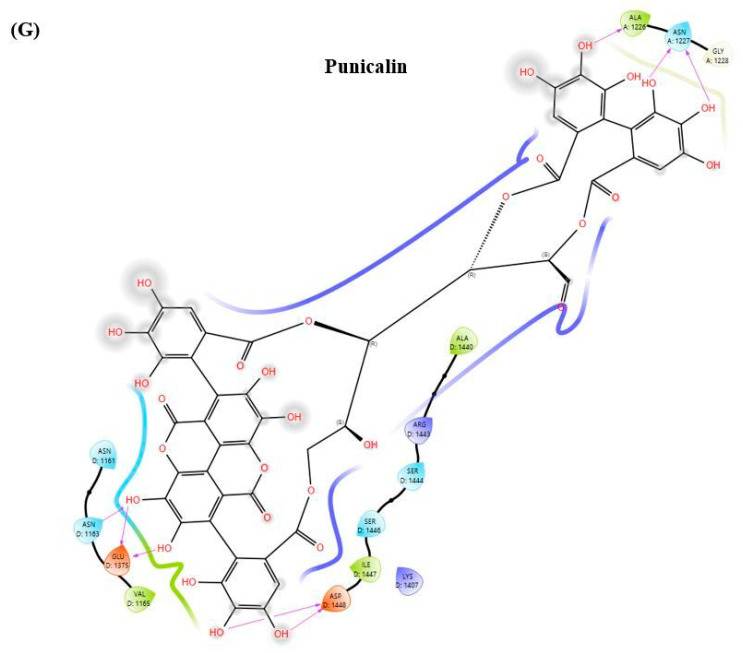
*Viroelixir* compounds docking with *Streptococcus mutans SpaP*. (**A**) Crystal structure of the C–terminal domain of the surface antigen SpaP from *S. mutans*. The protein is shown in ribbon (cartoon) representation with a color gradient indicating its structural organization. (**B**) Docking scores of *Viroelixir* compounds with SpaP. (**C**) Interaction of catechin with SpaP. (**D**) Interaction of ellagic acid with SpaP. (**E**) Interaction of caffeic acid with SpaP. (**F**) Interaction of epigallocatechin with SpaP. (**G**) Interaction of punicalin with SpaP. Red labels indicate oxygen atoms and hydroxyl (–OH) groups involved in hydrogen bonding interactions. Arrows represent hydrogen bonding and other non-covalent interactions between ligands and amino acid residues. Colored contours and lines indicate different interaction environments within the binding pocket.

To elucidate the molecular basis of *Viroelixir*’s anti-virulence activity, in silico docking analyses were performed between major phenolic constituents and three critical *S. mutans* virulence-associated proteins: glucosyltransferase B (GtfB), the LuxS quorum-sensing enzyme, and the surface adhesin SpaP. Docking simulations revealed stable and energetically favorable binding of multiple *Viroelixir* phenolics within the catalytic or functional domains of all three targets (Figure 6). Among them, punicalagin, catechin, epigallocatechin, ellagic acid, and caffeic acid consistently displayed the highest docking scores (Figure 7B, Figure 8B and Figure 9B).

For GtfB, several compounds occupied the catalytic pocket and interacted with conserved acidic residues (Asp354, Asp562, Asp567) and aromatic residues such as Trp491, which are essential for glucosyltransferase activity. These interactions were stabilized by extensive hydrogen-bonding networks and π–π stacking, suggesting steric and electrostatic interference with substrate binding and EPS synthesis.

Docking against LuxS revealed that most phenolics bound within the active site region involved in AI-2 synthesis, forming hydrogen bonds with key catalytic residues (Asp77, His58, Glu122) and hydrophobic interactions with surrounding residues. These binding modes are consistent with potential inhibition of quorum-sensing signaling pathways.

Similarly, docking to the C-terminal domain of SpaP demonstrated stable ligand accommodation along the adhesion interface, mediated by hydrogen bonding with Glu1375 and Asp1448 and supportive hydrophobic contacts. These interactions suggest a direct impairment of SpaP-mediated bacterial adhesion.

Docking analyses suggest potential molecular interactions between selected *Viroelixir* phenolic compounds and key virulence-associated proteins. These findings provide predictive insight rather than definitive mechanistic proof and require further experimental validation through enzyme inhibition assays and binding studies.

#### 2.6.1. Interaction of *Viroelixir* Components with GtfB

Based on the catalytic domain structure of glucosyltransferase B (*GtfB*) (Figure 7A,D), molecular docking analyses revealed that all major *Viroelixir* phenolics interact favorably with *GtfB*, displaying docking scores ranging from −5.332 to −9.942 (Figure 7B). Among the tested compounds (Figure 7C), punicalagin exhibited the strongest binding affinity (−9.942), followed by punicalin (−6.237), epigallocatechin (−5.990), catechin (−5.673), ellagic acid (−5.673) and caffeic acid (−5.541).

Overall, docking poses showed that *Viroelixir* components consistently occupy the *GtfB* catalytic pocket and interact with residues essential for enzymatic activity. Binding was predominantly stabilized by extensive hydrogen-bonding networks involving conserved acidic residues (Asp354, Asp562, Asp567), complemented by π–π stacking interactions with Trp491 and hydrophobic contacts with residues such as Leu356, Leu407, Tyr404, and Tyr584. These interactions position the ligands in close proximity to the catalytic region, suggesting potential interference with substrate binding and glucan synthesis.

Among the compounds, Punicalin exhibits tight binding within the active site via a dense hydrogen-bonding network involving Asp562, Asp567, Asp454, Arg570, Glu489, and Asn455, together with π–π interactions with Trp491 and Tyr404 and hydrophobic contacts with Leu356, Leu407, and Phe881 (Figure 7E). This binding mode is compatible with effective steric hindrance of substrate access.

Epigallocatechin binds within the GtfB catalytic cavity through extensive hydrogen bonds involving Asp562, Asp567, Glu564, Ser563, and Asn511, reinforced by π–π interactions with Trp491 and hydrophobic contacts with Leu407, Leu515, Tyr404, and Tyr584 (Figure 7F). This multivalent binding likely restricts substrate accessibility and catalytic turnover.

Catechin adopts a stable binding conformation supported by hydrogen bonding with Asp562 and Glu489, π–π stacking with Trp491, and hydrophobic interactions with Tyr404, Leu356, Leu407, and Phe881 (Figure 7G), suggesting effective anchoring within the catalytic region.

Ellagic acid is stabilized within the active site by strong hydrogen bonds with Asp562 and Ser563, supplemented by water-mediated interactions, π–π stacking with Trp491, and hydrophobic contacts involving Leu407, Tyr404, and Gly403 (Figure 7H). These interactions secure the rigid aromatic scaffold in a position likely to obstruct substrate binding.

Finally, caffeic acid binds through hydrogen bonding between its catechol hydroxyl groups and Asp562, electrostatic interactions involving its carboxylate moiety and Lys951, and π–π interactions with Trp491, supported by hydrophobic contacts with Leu356, Leu407, and Tyr404 (Figure 7I).

Collectively, these docking results suggest that *Viroelixir* phenolics engage *GtfB* through conserved and functionally relevant interactions, providing a molecular basis for the observed inhibition of EPS synthesis and biofilm matrix formation.

#### 2.6.2. Interaction of *Viroelixir* Components with LuxS Virulence Gene

Based on the crystal structure of the LuxS quorum-sensing enzyme (Figure 8A), molecular docking analyses demonstrated that 7 out of the 8 *Viroelixir* phenolic compounds interact favorably with LuxS, exhibiting docking scores ranging from −3.466 to −6.949 (Figure 8B). Among these compounds, punicalgin showed the strongest predicted binding affinity (−6.029), catechin (−5.925), caffeic acid (−5.547), ellagic acid (−5.030), and epigallocatechin (−3.466). Punicalgin could not be accommodated within the LuxS binding pocket due to steric constraints.

Overall, docking poses revealed that *Viroelixir* components preferentially occupy the *LuxS* catalytic region, engaging residues essential for AI-2 synthesis. Ligand binding was stabilized by extensive hydrogen bonding with conserved catalytic and polar residues, including Asp77, His58, Glu122, and Ser79, complemented by electrostatic interactions involving Lys23 and Arg20 and hydrophobic contacts with residues lining the active-site pocket, such as Ile76, Ile78, Phe7, Tyr88, Ala61, and Val35. These interactions suggest potential interference with LuxS catalytic activity and quorum-sensing signaling.

Among the compounds, Catechin binds stably within the *LuxS* catalytic cavity through extensive hydrogen bonding with Asp77, His58, Glu122, and Ser79, reinforced by a water-mediated interaction with Glu122 (Figure 8C). Electrostatic interaction with Lys23 and hydrophobic contacts involving Ile78, Leu59, Ala61, Val35, Phe7, and Tyr88 further contribute to ligand stabilization and optimal positioning.

Ellagic acid is accommodated within the *LuxS* active site through a dense network of hydrogen bonds involving Asp77, His58, Glu57, and Ser79, supported by water-mediated interactions near His58 (Figure 8D). Electrostatic interaction with Lys23 anchors the negatively polarized oxygen atoms, while hydrophobic residues (Ile76, Ile78, Val35, Tyr88, Ala61, and Pro121) stabilize the rigid phenolic scaffold.

Caffeic acid binds within the *LuxS* active pocket via hydrogen bonding between its phenolic hydroxyl groups and Asp77 and Ser79, complemented by electrostatic interaction between its carboxylate moiety and Arg20 (Figure 8E). Additional hydrogen bonding with Phe7 and Ser6, together with π–π stacking with Tyr88 and hydrophobic contacts involving Ile78, Ala61, Val9, and Ala8, further stabilizes the complex.

Epigallocatechin displays extensive binding within the *LuxS* active-site region, driven by multiple hydrogen bonds with Asp77, Ser79, His58, Glu57, Glu122, and Asp66, as well as several water-mediated interactions near Glu122 (Figure 8F). Electrostatic interactions with Arg65, Arg20, and Lys23, combined with hydrophobic contacts involving Ile76, Ile78, Ala61, Phe7, and Tyr88, contribute to strong ligand anchoring within the catalytic groove.

Collectively, these docking results indicate that *Viroelixir* phenolics can directly engage the *LuxS* catalytic site through conserved and functionally relevant interactions, providing a molecular basis for the observed inhibition of AI-2–mediated quorum sensing and the downregulation of *LuxS*-associated virulence pathways.

#### 2.6.3. Interaction of *Viroelixir* Components with SpaP Virulence Gene

Based on the crystal structure of the C-terminal domain of the surface adhesin SpaP (Figure 9A), molecular docking analyses demonstrated that 7 out of the 8 *Viroelixir* phenolic compounds interact favorably with *SpaP*, with docking scores ranging from −3.022 to −6.556 (Figure 9B). Among these compounds, catechin (−6.555), and punicalin (−6.029) exhibited the strongest predicted binding affinities, followed by ellagic acid (−5.523), caffeic acid (−5.375), and epigallocatechin (−3.022).

Overall, docking poses revealed that *Viroelixir* components bind within the SpaP adhesion groove via conserved, functionally relevant interactions. Ligand stabilization was primarily driven by extensive hydrogen bonding with acidic residues Glu1375 and Asp1448, supported by polar contacts with Asn1161 and Asn1163 and hydrophobic interactions involving Val1165 and Ile1447. Electrostatic contributions from Lys1407 further enhanced ligand anchoring in several complexes. These interactions suggest that *Viroelixir* phenolics may directly interfere with *SpaP*-mediated adhesion.

Among the compounds, Catechin adopts a well-defined binding conformation driven by hydrogen bonding with Glu1375, Asp1448, and Lys1407, reinforced by polar contacts with Asn1161 and Asn1163 (Figure 9E). Hydrophobic interactions with Val1165 and Ile1447 and polar support from Ser1446 further stabilize the ligand within the adhesion groove.

Ellagic acid binds firmly within the *SpaP* pocket through strong hydrogen bonds with Glu1375 and Asp1448, supplemented by additional interactions with Ser1446 and electrostatic stabilization via Lys1407 (Figure 9F). Hydrophobic contacts involving Val1165 and Ile1447 contribute to stabilization of the rigid phenolic scaffold.

Caffeic acid is accommodated within the *SpaP* binding region through hydrogen bonding of its carboxylate and hydroxyl groups with Asn1161, Asn1163, Glu1375, and Asp1448 (Figure 9G). These interactions are reinforced by hydrophobic contacts with Val1165 and Ile1447 and polar support from Ser1446 and Lys1407.

Epigallocatechin displays a dense interaction network dominated by hydrogen bonding with Lys1407, Glu1375, and Asp1448, supported by polar contacts with Asn1408 and Asn1163 and hydrophobic interactions with Val1165, Ile1447, and Tyr1152 (Figure 9F). This multivalent binding suggests high complementarity within the *SpaP* binding pocket.

Finally, punicalin exhibits extensive polar and hydrophobic interactions along the *SpaP* surface (Figure 9G). Multiple hydroxyl groups form hydrogen bonds with Asn1227, Asn1161, Asn1163, Gly1228, Glu1375, and Asp1448, while additional stabilization is provided by interactions with Ser1444, Ser1446, Arg1443, and hydrophobic residues such as Ala1226, Ala1440, Val1165, and Ile1447. The extended aromatic scaffold allows cooperative interactions across the binding groove, consistent with strong affinity and effective *SpaP* inhibition.

Collectively, these docking results suggest that *Viroelixir* phenolics engage *SpaP* through conserved adhesion-related residues, providing a molecular basis for impaired bacterial attachment and reduced virulence observed experimentally.

### 2.7. Evaluation of Cytotoxicity on Human Gingival Epithelial Cells

**Figure 10:**
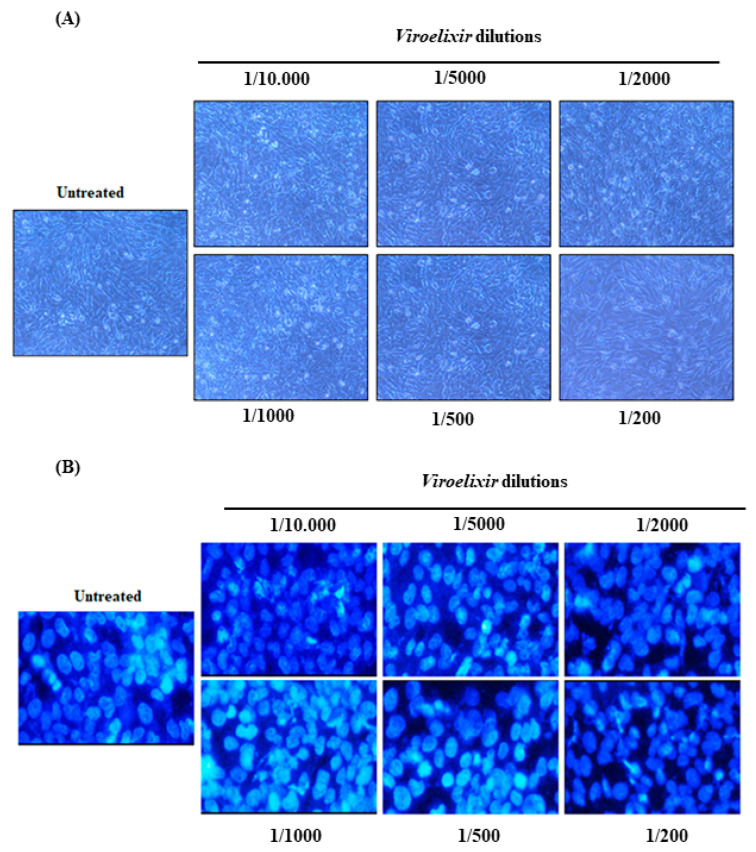

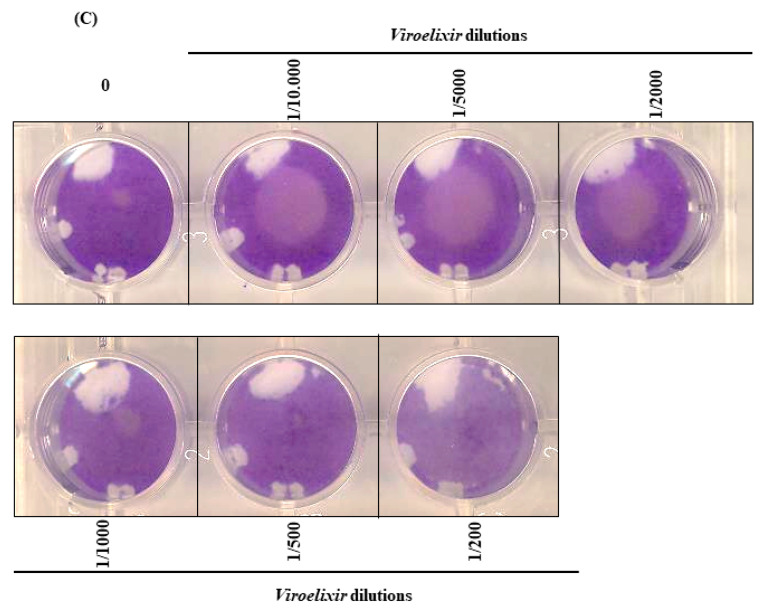
Evaluation of the cytotoxic effects of *Viroelixir* on human gingival epithelial cells. (**A**) Representative images (*n* = 4) taken after 24 h of exposure to increasing dilutions of *Viroelixir* (1/10,000 to 1/50), showing normal cell morphology, adhesion, and confluence with no detectable alterations. (**B**) Hoechst staining (*n* = 4), evaluating nuclear integrity under the same conditions. (**C**) Crystal violet staining (*n* = 4) assessing cell morphology after 24 h of exposure to increasing dilutions of *Viroelixir* at dilutions ranging from 1/10,000 to 1/200. No cytotoxic effects were observed across the tested concentrations. Data represent the mean ± SD of four independent experiments.

The cytotoxicity of *Viroelixir* was assessed in human gingival epithelial cells (GMSMK) using morphological evaluation, Hoechst nuclear staining, and crystal violet staining. As shown in Figure 10A–C, *Viroelixir* exhibited no detectable cytotoxicity at any tested dilution. Phase-contrast microscopy (Figure 10A) revealed that cell morphology, adhesion, and confluence remained indistinguishable from those of untreated controls, with no evidence of shrinkage, detachment, or structural alterations. Hoechst staining (Figure 10B) suggested nuclear integrity, showing uniformly stained, round nuclei without chromatin condensation or fragmentation, indicative of the absence of apoptotic or necrotic processes. Crystal violet staining (Figure 10C) further supported these findings, showing comparable staining intensity and cell coverage among all conditions, reflecting unchanged viability and proliferation. Collectively, these results demonstrate that *Viroelixir* is non-cytotoxic to oral epithelial cells, supporting its suitability for therapeutic or preventive dental applications.

## 3. Discussion

This present study suggests that *Viroelixir* exerts potent antibacterial and anti-virulence effects on *S. mutans* while remaining non-toxic to oral epithelial cells. By integrating microbiological, transcriptional, and in silico docking analyses, our data provide mechanistic insights into how *Viroelixir* attenuates carcinogenicity through simultaneous targeting of bacterial growth, metabolism, quorum sensing, adhesion, and biofilm formation.

Chemical characterization by LC–MS revealed that *Viroelixir* contains a complex mixture of phenolic compounds, dominated by two major chromatographic peaks and a wide molecular mass distribution. This chemical diversity is consistent with the presence of green tea catechins and pomegranate-derived ellagitannins, compounds known for their antimicrobial and anti-inflammatory activities. Green tea catechins, particularly EGCG, EGC, ECG, and EC, are relatively polar compounds that elute within comparable retention times under reversed-phase LC conditions and exhibit intense UV absorption. Their molecular masses fall within the lower region of the observed mass spectrum, making them strong candidates for one of the dominant peaks. Pomegranate-derived phytochemicals, especially ellagitannins such as punicalagin, ellagic acid derivatives, and galloylated phenolics, also display strong UV absorbance and produce MS fragmentation patterns compatible with the TIC profile. It is therefore plausible that the second major peak represents an ellagitannin or related galloylated phenolic acid. Together, these analytical observations suggest that *Viroelixir*’s biological effects arise from the combined activity of green tea catechins and pomegranate ellagitannins acting in a complementary or synergistic manner. Although targeted MS/MS analysis will be required for definitive phenolic compound identification, the current data strongly support the interpretation that these two phytochemical families are the main contributors to the extract’s potent antibacterial and anti-virulence activities. Their presence correlates directly with the phenotypic and transcriptional effects observed in *S. mutans* throughout this study.

The antibacterial activity observed is consistent with known effects of phenolics, including membrane disruption, interference with metabolic enzymes, and metal ion chelation. The strong reduction in acid production suggests impairment of glycolytic flux, potentially through inhibition of enolase or lactate dehydrogenase. Phenolic compounds are known to alter membrane permeability, inhibit energy metabolism, and interfere with protein synthesis in Gram-positive bacteria [40,41,43]. Catechins, particularly epigallocatechin-3-gallate (EGCG) and epicatechin-3-gallate (ECG), can penetrate Gram-positive bacterial cell walls and disrupt the cytoplasmic membrane, leading to leakage of intracellular ions, proteins, and nucleotides [40] Ellagic acid and hydrolysable tannins complement these effects by binding to cell wall proteins, restricting nutrient transport, and inducing mild oxidative stress [41] within bacterial cells, leading to damage of DNA, proteins, and lipids, further contributing to cell death [36,37,43]. These combined mechanisms may explain the delayed regrowth of *S. mutans* observed at sub-MIC concentrations.

Green tea catechins such as EGCG have been shown to inhibit *S. mutans* glucosyltransferase activity, reduce biofilm formation, and cause leakage of intracellular components [40,43]. Similarly, pomegranate ellagitannins disrupt bacterial membranes and inhibit metabolic enzymes [41,42]. Acid production by *S. mutans* is a major virulence determinant that leads to enamel demineralization [7,8]. The observed reduction in ΔpH following *Viroelixir* treatment indicates an inhibition of glycolytic metabolism and lactic acid synthesis [34,36,43].

Biofilm formation is a key virulence trait of *S. mutans*, conferring protection against environmental stress and antimicrobial agents [10,11]. Quantitative and ultrastructural analyses demonstrated that *Viroelixir* markedly reduced biofilm biomass and disrupted biofilm architecture. These phenotypic effects were supported at the transcriptional level by significant downregulation of *gtfB* and *gbpB*, genes encoding enzymes essential for extracellular polysaccharide (EPS) synthesis and biofilm matrix stabilization [10,11]. Importantly, the docking analyses provide a molecular rationale for these observations. *Viroelixir* phenolics displayed strong and stable binding within the catalytic pocket of glucosyltransferase B (*GtfB*), engaging conserved acidic residues (Asp354, Asp562, Asp567) and aromatic residues such as Trp491 that are critical for enzymatic activity. These interactions suggest direct steric and electrostatic interference with substrate binding and glucan synthesis, thereby impairing EPS production and biofilm maturation.

Quorum sensing plays a central role in coordinating *S. mutans* virulence, competence development, and stress adaptation [12,13,14,15,16,17,18]. In this study, *Viroelixir* significantly downregulated quorum-sensing–related genes, including *comR*, *comD*, *comX*, *luxS*, and *nlmD*. Docking analyses further revealed that multiple *Viroelixir* phenolics bind favorably within the active site of *LuxS*, interacting with key catalytic residues such as Asp77, His58, and Glu122. These findings suggest that *Viroelixir* may directly inhibit AI-2 synthesis, thereby disrupting *LuxS*-mediated intercellular communication. Such interference with quorum sensing likely contributes to the observed suppression of virulence gene expression and biofilm development.

Adhesion to the tooth surface is an early and critical step in *S. mutans* colonization and the establishment of cariogenic biofilms [19,20,21,22,23,24]. The major surface adhesin *SpaP* (AgI/II) mediates sucrose-independent attachment and is strongly associated with caries risk [24]. In the present study, *spaP* expression was markedly downregulated following *Viroelixir* treatment. Consistently, docking analyses showed that several *Viroelixir* phenolics bind along the SpaP adhesion groove through conserved interactions with acidic residues Glu1375 and Asp1448, supported by polar and hydrophobic contacts. These interactions suggest direct impairment of *SpaP*-mediated adhesion, providing a mechanistic explanation for reduced biofilm formation and bacterial attachment observed experimentally.

Although docking simulations suggest plausible interactions between *Viroelixir* phenolics and virulence-associated proteins, these in silico observations remain predictive and hypothesis-generating. Biochemical and biophysical validation studies will be necessary to establish direct enzymatic inhibition and binding specificity. Stress tolerance is another critical factor contributing to *S. mutans* persistence in the acidic and nutrient-limited oral environment [25]. The observed downregulation of *relA* and *relP* indicates that *Viroelixir* may impair the stringent response, weakening bacterial survival under adverse conditions. This effect likely acts synergistically with the inhibition of quorum sensing and EPS synthesis to further attenuate virulence.

Importantly, unlike conventional antiseptics such as chlorhexidine, which may induce cytotoxicity and disrupt oral microbial homeostasis [28]. *Viroelixir* exhibited no detectable cytotoxic effects on human gingival epithelial cells. This favorable biocompatibility profile supports its potential for long-term use in preventive and restorative dental applications.

Taken together, our findings indicate that *Viroelixir* acts through a multi-target anti-virulence mechanism, combining (i) inhibition of bacterial growth and acid production [40,41], (ii) transcriptional suppression of key virulence genes [40,43], (iii) direct molecular interference with essential virulence proteins, including *GtfB*, *LuxS*, and *SpaP* [12,15,17]. and weakening of stress response pathways [25]. These multi-target effects reduce the probability of resistance development and highlight the potential of *Viroelixir* as a safe, natural antimicrobial for caries prevention.

Several limitations should be acknowledged. First, LC–MS compound identification remains tentative, as targeted MS/MS fragmentation and calibration with authentic standards were not performed. Second, quantitative profiling of individual phenolic compounds was not conducted. Third, docking analyses are predictive and hypothesis-generating in nature and require biochemical validation. Finally, in vitro findings may not fully replicate the complexity of the oral microbiome environment.

While phenotypic assays provide direct evidence of anti-virulence activity, docking analyses serve to generate mechanistic hypotheses regarding potential ligand–protein interactions. These computational findings should be interpreted cautiously and validated experimentally. Future studies including enzymatic inhibition assays and direct binding validation will be necessary to confirm these predicted interactions

## 4. Materials and Methods

### 4.1. Materials

#### 4.1.1. Bacterial Strain and Growth Conditions

*S. mutans* (ATCC 25175) obtained from the American Type Culture Collection (Manassas, VA, USA) was cultured in Brain Heart Infusion (BHI) broth (Multicell, Wisent Inc., Quebec City, QC, Canada) prepared according to the manufacturer’s instructions. Media were sterilized by autoclaving at 121 °C for 15 min and stored at 4 °C until use. Before inoculation, the media were prewarmed to room temperature.

Methanol (HPLC-grade, ≥99.9%) and formic acid (LC–MS grade) were purchased from Sigma-Aldrich (St. Louis, MO, USA).

#### 4.1.2. Preparation of *Viroelixir*

*Viroelixir*, a natural phenolic extract provided by Dr. Gin Wu (Comcast, Taiwan). *Viroelixir* was prepared by suspending 5 g of green tea leaves and 5 g of pomegranate peel powder in 100 mL of sterile distilled water. The mixture was stirred continuously for 24 h at room temperature under light-protected conditions to allow aqueous extraction of phenolic compounds. After extraction, the suspension was filtered through sterile 2 μm filters to remove plant debris. The filtrate was collected as a stock solution and stored at 4 °C until use. Working dilutions (1/10,000 to 1/50) were freshly prepared before each experiment. Penicillin–streptomycin (Gibco, Thermo Fisher Scientific, Waltham, MA, USA) was dissolved in sterile distilled water according to the manufacturer’s instructions. Penicillin–streptomycin (P/S) (final concentration: 0.2 IU/mL penicillin and 0.05 µg/mL streptomycin) served as a positive control.

### 4.2. Methods

#### 4.2.1. Liquid Chromatography–Mass Spectrometry (LC–MS) Analysis

The phytochemical composition of *Viroelixir* was analyzed using high-performance liquid chromatography coupled with high-resolution mass spectrometry (LC–HRMS). Chromatographic separation was performed on an Altima C18 reverse-phase column (5 µm, 4.6 × 250 mm). The mobile phase consisted of solvent A (water containing 0.1% formic acid) and solvent B (methanol containing 0.1% formic acid). The gradient program was as follows: 0–5 min, 90% A/10% B; 5–30 min, linear gradient to 100% B; 30–40 min, 100% B (column washing); and 40–50 min, re-equilibration to initial conditions. The flow rate was maintained at 0.8 mL/min, and the injection volume was 10 µL.

High-resolution mass spectrometry analysis was performed using a quadrupole time-of-flight (Q-TOF) mass spectrometer equipped with an electrospray ionization source operated in negative ion mode (ESI−), which is suitable for the detection of phenolic compounds. The capillary voltage was set at 3.5 kV, the source temperature at 120 °C, and the desolvation temperature at 350 °C. Mass spectra were acquired over an *m*/*z* range of 50–1500. External mass calibration was performed prior to analysis, and mass accuracy was maintained below 5 ppm.

All LC–HRMS analyses were carried out at the analytical platform of the Department of Chemistry, Université Laval (Quebec City, QC, Canada). Because analyses were conducted through a centralized analytical facility, detailed resolving power and calibration specifications follow the standard operating procedures of the platform.

Compound identification was considered tentative and based on accurate mass measurements, chromatographic behavior, and comparison with previously reported phytochemical profiles and literature data.

#### 4.2.2. Bacterial Growth Inhibition Assay

To determine the bacteriostatic activity of *Viroelixir*, *S. mutans* (10^5^ CFU/mL) was cultured in BHI broth containing different *Viroelixir* dilutions (1/10,000–1/50). Bacterial growth was monitored by measuring the optical density (OD) at 660 nm every two hours for 24 h using a Synergy 2 microplate reader (BioTek Instruments, Winooski, VT, USA) [45]. The Minimum Inhibitory Concentration (MIC) was defined as the lowest dilution preventing visible growth. (P/S) was used as a positive control. Results were expressed as the percentage of proliferation relative to untreated cells (100%).

#### 4.2.3. BHI Agar Test

The Minimum Bactericidal Concentration (MBC) of *Viroelixir* was evaluated using a plate-counting method. Aliquots (50 µL) of *S. mutans* suspension (10^4^ CFU/mL), treated or not with *Viroelixir*, were spread on BHI agar (Multicell, Wisent Inc., Quebec City, QC, Canada) supplemented with 12% defibrinated sheep blood (Nutri-Bact, Terrebonne, QC, Canada). The plates were incubated at 30 °C under 5% CO_2_ for 24 h. The MBC was defined as the lowest concentration at which no visible colonies were detected. Plates were scanned using an HP Scanjet 4070 Photosmart Scanner (Hewlett-Packard, Palo Alto, CA, USA). All experiments were performed independently in triplicate.

#### 4.2.4. Crystal Violet Assay

Biofilm formation was assessed by crystal violet staining. *S. mutans* (10^5^ CFU/mL) was incubated for 6 h and 24 h with or without *Viroelixir*. Pelleted cells were washed twice with phosphate-buffered saline 1X (PBS 1X), resuspended in 100 µL of 1% crystal violet solution Crystal violet (≥90%, Sigma-Aldrich, St. Louis, MO, USA, CAS No. 548-62-9), and stained for 10 min at room temperature. After washing and drying overnight, the stained pellets were solubilized in 500 µL of 30% acetic acid (analytical grade, Sigma-Aldrich, St. Louis, MO, USA). Absorbance was measured at 570 nm. Results were expressed as mean ± standard deviation (SD) from seven independent assays.

#### 4.2.5. pH Drop (Acidogenicity) Test

To assess the effect of *Viroelixir* on acid production, *S. mutans* (10^6^ CFU/mL) cultures were incubated with various *Viroelixir* dilutions for 24 h at 37 °C. After incubation, the bacterial suspensions were centrifuged at 1000 rpm for 5 min, and the pH of the supernatants was measured using universal pH indicator strips (MColorpHast™, Millipore, Burlington, MA, USA). The pH change (ΔpH) was calculated as ΔpH = pH (final) − pH (initial); P/S served as a positive control. Each experiment was repeated independently four times.

#### 4.2.6. Hemolytic Activity Assay

The hemolytic potential of *S. mutans* treated with *Viroelixir* was evaluated according to Vaillancourt et al. (2022) [46]. Sheep blood was centrifuged at 600× *g* for 5 min, and erythrocytes were washed in PBS ×1 and resuspended to 2% (*v*/*v*). Equal volumes of erythrocyte suspension and *S. mutans* treated with *Viroelixir* were incubated overnight at 37 °C, followed by 1 h at 4 °C. After centrifugation (10,000× *g*, 5 min), supernatant absorbance was measured at 540 nm. Percent hemolysis was normalized to the untreated bacterial control (100%).

#### 4.2.7. Mature Biofilm Formation and Scanning Electron Microscopy (SEM)

Mature biofilms were developed by seeding 10^6^ CFU/mL of *S. mutans* onto 5 × 5 mm CollaTape matrices (Zimmer Dental, Carlsbad, CA, USA) and culturing for six days in BHI medium containing different *Viroelixir* dilutions. The medium was refreshed every two days. Biofilms treated with P/S (5 µg/mL) served as positive controls. After fixation in 4% paraformaldehyde for 20 min, samples were processed for SEM imaging as described previously in our studies [47,48]. Three independent experiments were performed.

#### 4.2.8. RT-qPCR Analysis of Virulence Gene Expression

The influence of *Viroelixir* on the expression of virulence genes (*comD*, *comR*, *comX*, *gbpB*, *gtfB*, *luxS*, *nlmD*, *relA*, *relP*, and *spaP*) was analysed by quantitative RT-PCR. *S. mutans* (10^7^ CFU/mL) were cultured for 24 h in the absence or presence of *Viroelixir* (1/1000 and 1/200 dilutions). The 1/1000 dilution was chosen as the lowest concentration at which an antibacterial effect began to appear, while the 1/200 dilution corresponded to the half-maximal inhibitory concentration (IC_50_), ensuring sufficient bacterial growth for RNA extraction and accurate expression analysis. Total RNA was extracted using standard phenol–chloroform methods and reverse-transcribed into cDNA using the iScript™ cDNA Synthesis Kit (Bio-Rad, Saint-Laurent, QC, Canada). qPCR was performed using iQ™ SYBR^®^ Green Supermix (Bio-Rad). Specific primer sequences used for qPCR are listed in Table 1, and the 16S rRNA gene served as the housekeeping control, and relative expression was calculated using the 2^−ΔΔCt^ (Livak) method.

#### 4.2.9. Docking Study

##### Ligand Preparation

Computational studies were performed using a curated ligand library prepared with the LigPrep module (Schrödinger Release 2025-2, Schrödinger, LLC, New York, NY, USA). Ligands were processed using the OPLS_2005 force field, and all possible ionization states were generated at pH 7.0 ± 2.0 using Epik, while retaining the original chemical state of each ligand. Stereochemistry was preserved where specified, and energetically minimized three-dimensional conformations were generated for subsequent docking studies.

##### Protein Preparation

Crystal structures of the target proteins—LuxS quorum-sensing protein (PDB ID: 5E68), SpaP surface antigen (PDB ID: 3OPU), and GtfB (PDB ID: 8FG8)—were retrieved from the RCSB Protein Data Bank. Protein preparation was carried out using the Protein Preparation Wizard in Maestro 14.4 (Schrödinger 2025-2). The preparation protocol included bond order assignment, addition of hydrogen atoms, removal of crystallographic water molecules, optimization of hydrogen-bonding networks, and restrained energy minimization using the OPLS_2005 force field. All ionizable residues were adjusted to pH 7.0, and structures were converted from 2D to optimized 3D conformations.

##### Active Site Prediction

The active sites of LuxS, SpaP, and GtfB were identified using the SiteMap module or by generating receptor grids based on co-crystallized ligands, where available. SiteMap analysis was performed to locate energetically favorable ligand-binding regions on the protein surface by evaluating pocket size, enclosure, hydrophobicity, and hydrogen-bonding capability. Grid boxes were generated around the predicted binding sites using Maestro 14.4 (Schrödinger 2025-2).

##### Glide Docking

Prepared ligands and reference co-crystallized compounds were docked into the active sites of the target proteins using the Glide docking module in Maestro 14.4 (Schrödinger 2025-2). Docking was performed using the Standard Precision (SP) mode with flexible ligand sampling. Default docking parameters recommended by Schrödinger were applied. Binding poses were ranked based on GlideScore (G-score) and docking score, and the most energetically favorable conformations were selected for interaction analysis.

#### 4.2.10. Gingival Epithelial Cell Growth Assay

Human gingival epithelial cells (GMSMK) provided by Dr. Daniel Grenier (GREB, Laval University, Quebec, QC, Canada) were seeded (1 × 10^5^ cells/well) in 24-well plates containing Dulbecco’s Modified Eagle Medium (DMEM) (Gibco, Thermo Fisher Scientific, Waltham, MA, USA) supplemented with 10% fetal bovine serum (FBS) (Thermo Fisher Scientific, Waltham, MA, USA). After 24 h of adhesion, cells were treated with different *Viroelixir* dilutions for 24 h at 37 °C in 5% CO_2_. Cells were fixed with methanol (Fisher Scientific, Ottawa, ON, Canada), stained with 1% crystal violet (Sigma-Aldrich, St. Louis, MO, USA), and imaged under a Leica optical microscope (Leica Microsystems, Concord, ON, Canada). Absorbance was measured at 570 nm using a Bio-Rad xMark™ Microplate Spectrophotometer (Bio-Rad Laboratories, Hercules, CA, USA) to evaluate cell proliferation.

#### 4.2.11. Hoechst Staining for Nuclear Integrity

To assess potential nuclear alterations, GMSMK cells grown on sterile glass coverslips were treated with *Viroelixir* for 24 h, fixed with methanol/acetic acid (75:25 *v*/*v*), and stained with Hoechst 33,342 (Thermo Scientific, Waltham, MA, USA) in the dark at room temperature. Samples were examined under a Nikon fluorescence microscope equipped with a DAPI filter. Representative fluorescent images were captured from three independent experiments (*n* = 3).

#### 4.2.12. Statistical Analysis

All data are presented as mean ± standard deviation (SD). Statistical differences between control and treated groups were assessed using one-way analysis of variance (ANOVA) followed by Tukey’s post hoc test or Student’s *t*-test, as appropriate. A *p* < 0.05 was considered statistically significant (*p* < 0.05, * *p* < 0.01, ** *p* < 0.001). Analyses were performed using GraphPad Prism (version 8.4).

## 5. Conclusions

Collectively, these findings suggest that *Viroelixir* may act as a multi-target phenolic formulation capable of attenuating key virulence determinants of *S. mutans*. Further mechanistic and translational studies are warranted to confirm its therapeutic potential.

## Figures and Tables

**Table 1:** Primer sequences used for qPCR.

*S. mutans* Genes	Primer Sequence	Amp Size(bp)
*ComX*	5′-CGTCAGCAAGAAAGTCAGAAAC-3′ 3′-ATACCGCCACTTGACAAACAG-5′	89
*ComD*	5′-TATGGTCTCTGCCTGTTGC-3′ 3′-TGCTACTGCCCATTACAATTCC-5′	97
*ComR*	5′-TATTACGAAGGCCAACCTAT-3′ 3′-TTCTTCTTCAGGCAAATCAT-5′	C 104
*nlmD*	5′-TGGTGGTATGATTAGATGTGCACTTGG-3′ 3′-CTAAAGCCGCTCCAGATACTGTACC-5′	122
*Spap*	5′-TTGCCGATGAAACGACCACTAC-3′ 3′-TCAGCTTCCTTACTCGCACTCC-5′	115
*LuxS*	5′-ACTGTTCCCCTTTTGGCTGTC-3′ 3′-AACTTGCTTTGATGACTGTGGC-5′	93
*RelA*	5′-CGCIGAGGCATTTACGCAAGG-3′ 3′-GCGACTAATCCCCAGCCGATG-5′	94
*Rel P*	5′-AGACACGCCATTTGAGGATTGC-3′ 3′-GGTGCTCCAAACTAGCCCAGG-5′	104
*gbpB*	5′-AGCAACAGAAGCACAACCATCAG-3′ 3′-CCACCATTACCCCAGTAGTTTCC-5′	150
*gtfB*	5′-ACACTTTCGGGTGGCTTG-3′ 3′-GCTTAGATGTCACTTCGGTTG-5′	127
*16S rRNA*	5′-CTTACCAGGTCTTGACATCCCG-3′ 3′-ACCCAACATCTCACGACACGAG-5′	113

## Data Availability

All data generated or analyzed during this study are included in this published article.

## Abbreviations

AgI/II	Antigen I/II (SpaP adhesin)
ANOVA	Analysis of Variance
ATCC	American Type Culture Collection
BHI	Brain Heart Infusion
cDNA	Complementary DNA
CFU	Colony-Forming Units
ComDE	Two-Component Regulatory System ComDE
ComRS	ComRS Regulatory System
ComX	Competence Sigma Factor X
CSP	Competence-Stimulating Peptide
DAPI	4′,6-Diamidino-2-Phenylindole
DMEM	Dulbecco’s Modified Eagle Medium
ECG	Epicatechin-3-Gallate
EGCG	Epigallocatechin-3-Gallate
EPS	Extracellular Polymeric Substance
FBS	Fetal Bovine Serum
GbpB	Glucan-Binding Protein B
GMSMK	Human Gingival Epithelial Cells (GMSMK line)
Gtf/GtfB	Glucosyltransferase/Glucosyltransferase B
GC–MS	Gas Chromatography–Mass Spectrometry
IC_50_	Half-Maximal Inhibitory Concentration
LC–MS	Liquid Chromatography–Mass Spectrometry
luxS	*luxS* quorum-sensing gene
MBC	Minimum Bactericidal Concentration
MIC	Minimum Inhibitory Concentration
nlmD	Mutacin-related gene *nlmD*
OD	Optical Density
PBS	Phosphate-Buffered Saline
P/S	Penicillin–Streptomycin
qPCR	Quantitative Polymerase Chain Reaction
relA, relP, relQ	(p)ppGpp synthetases
RT-qPCR	Reverse Transcription Quantitative Polymerase Chain Reaction
*S. mutans*	*Streptococcus mutans*
SD	Standard Deviation
SEM	Scanning Electron Microscopy
SpaP	Surface Protein Antigen I/II (P1/AgI/II)
16S rRNA	16S Ribosomal RNA
